# CD59 is a potential biomarker of esophageal squamous cell carcinoma radioresistance by affecting DNA repair

**DOI:** 10.1038/s41419-018-0895-0

**Published:** 2018-08-30

**Authors:** Yuzhen Zhou, Li Chu, Qi Wang, Weixing Dai, Xiaozhou Zhang, Jianfeng Chen, Ling Li, Peipei Ding, Long Zhang, Hongyu Gu, Luying Li, Xinyue Lv, Wei Zhang, Danlei Zhou, Pingzhao Zhang, Guoxiang Cai, Kuaile Zhao, Weiguo Hu

**Affiliations:** 1 Fudan University Shanghai Cancer Center and Institutes of Biomedical Sciences, Shanghai Medical College, Fudan University, 200032 Shanghai, China; 2 Department of Radiation Oncology, Fudan University Shanghai Cancer Center, Shanghai Medical College, Fudan University, 200032 Shanghai, China; 3 Department of Colorectal Surgery, Shanghai Medical College, Fudan University, 200032 Shanghai, China

## Abstract

Radiation therapy is an important treatment modality for esophageal cancer. However, acquisition of radioresistance ultimately results in esophageal cancer relapse. CD59, a membrane-bound complement regulatory protein, can transduce signals via a Src kinase in the lipid raft, thus playing a complement-independent role. However, the effect of CD59 on the esophageal cancer response to ionizing radiation remains unclear. In this study, we found that the expression level of CD59 was positively correlated with the radioresistance of esophageal cancer cell lines and clinical specimens. High CD59 expression indicated poor overall survival (OS) and disease-free survival (DFS) in esophageal squamous cell carcinoma (ESCC) patients who received radiotherapy. Genetic alteration of CD59 expression modulated the radiosensitivity of esophageal cancer cells to ionizing radiation. CD59 deficiency exacerbated DNA damage, hindered cell proliferation, and induced G2/M cell cycle arrest and cellular senescence, leading to an impaired DNA damage repair ability. In addition, CD59 deficiency almost completely reduced the phosphorylation of Src at Y416 despite ionizing radiation. A Src inhibitor saracatinib sensitized esophageal cancer cells to irradiation. Therefore, CD59 may be a potential biomarker for predicting the radioresistance of ESCC to radiotherapy.

## Introduction

Esophageal cancer is ranked the eighth most aggressive cancer and the sixth most common cause of cancer-related deaths worldwide^1,2^. Esophageal cancer has a poor prognosis due to early metastasis, and the 5-year overall survival (OS) rate is <20%^3^. In 2011, the estimated numbers of new esophageal cancer cases and deaths were 291,238 and 218,957, respectively, in China from 177 cancer registries from 28 provinces^4^. Esophageal cancer is classified into two histological groups: esophageal squamous cell carcinoma (ESCC) and esophageal adenocarcinoma (EAC). ESCC is the predominant histologic subtype in China, where ESCC accounts for approximately 88.8% of all esophageal cancer cases^4^. Surgery remains the predominant treatment, particularly for early-stage esophageal cancer patients. However, most esophageal cancer patients are diagnosed after late-stage presentation. Thus, radiotherapy has become a widely used option for those patients with unresectable esophageal cancer.

Exposure to ionizing radiation may induce high levels of clustered DNA damage, including complex double-strand breaks (DSB), to destroy tumor cells because clustered DNA damage is difficult to repair^5,6^. For the maintenance of genomic integrity, the DNA damage response (DDR) is rapidly activated in response to DNA damage. This process initially involves the activation of either the serine/threonine protein kinases ataxia telangiectasia mutated (ATM), ataxia telangiectasia and Rad3-related or DNA-dependent protein kinase catalytic subunit, subsequently leading to the phosphorylation of histone H2AX at S139 (γH2AX)^7–11^. γH2AX largely forms at nascent DSB sites within 30 min, further generating γH2AX foci with the accumulation of proteins involved in DNA repair and chromatin remodeling^7,10–12^. Irreversible DNA damage leads to the induction of cellular senescence, mitotic catastrophe, necrosis and/or apoptosis^13^. Any disorder with such processes may result in radioresistance. Although the exact mechanism has not yet been elucidated, a disturbed DDR, increased basal activity of the DNA repair complex and abnormal activation of pro-survival and pro-proliferation signaling pathways commonly underlie radioresistance^14–21^. The acquisition of intrinsic and induced radioresistance leads to local recurrence and distant metastasis, which ultimately results in relapse and treatment failure^22^. Therefore, the identification of biomarkers to precisely predict radiosensitivity and the identification of additional targets and modalities for improving radiosensitivity are urgently needed for esophageal cancer treatment.

The immune system plays a dual role in cancer suppression and promotion due to the switch between immune surveillance and escape^23,24^. Similarly, the complement system, a key system for immune surveillance and homeostasis^25^, has also been reported to play a controversial role in radiotherapy. Irradiation results in tumor cell apoptosis and local complement activation in fractionated radiotherapy for lymphoma, and local complement inhibition markedly improves the therapeutic efficacy of radiotherapy due to enhanced apoptosis and inflammation^26^. In contrast, acute and transient local complement activation primarily improved the therapeutic efficacy of radiotherapy against murine and human tumors via C3a/C5a-activated tumor-specific immunity^27^.

CD59, a small glycosylphosphatidylinositol (GPI)-linked glycoprotein, is the sole membrane-bound complement regulatory protein (mCRP) that restricts the assembly of the membrane attack complex (MAC, C5b-9n) by binding to C8/C9^28,29^. CD59 is widely expressed on almost all host cells to prevent the inappropriate deposition of MAC^30^. However, tumor cells maliciously hijack CD59 to escape from complement immune surveillance^31,32^ and complement-dependent cytotoxicity (CDC) induced by anticancer antibodies^33,34^. In addition, many studies have attributed CD59 a complement-independent role in signaling transduction. Lipid rafts, which float in the bilayer of the plasma membrane, are composed of cholesterols, glycosphingolipids, sphingolipids, saturated phospholipids, and GPI-anchored proteins, in which CD59 has been widely accepted as a lipid raft marker^35–38^. Cross-linking of CD59 with other raft components leads to the formation of stabilized membrane patches enriched with Src kinase family proteins, which are thereby centers of signal transduction^39–43^. Numerous studies have demonstrated the deleterious effect of CD59 expression on hindering antibody-based cancer immunotherapy;^33,34^ however, a limited reports about the effect of CD59 on chemotherapy, which revealed that CD59 insufficiency sensitizes tumor cells to chemotherapy, likely due to the resultant pro-apoptotic effect^44,45^. Furthermore, there has been no report on the effect of CD59 on radiotherapy.

In this study, we demonstrated that CD59 deficiency sensitized ESCC cells to radiotherapy. We examined the relationship between CD59 expression and radioresistance and investigated the influence of CD59 deficiency on the radiosensitivity of esophageal cancer cell lines using various in vitro and in vivo assays. Furthermore, CD59 deficiency attenuated the activation of Src(Y416) and impaired the DNA damage repair and proliferation capacity, which resulted in accumulated γH2AX foci. CD59 deficiency caused prolonged G2/M cell cycle arrest and ultimately resulted in the senescence of ESCC cells. The Src inhibitor saracatinib sensitized ESCC cells to irradiation. This study showed the complement-independent role of CD59 and revealed novel therapeutic opportunities for improving the radiation response of esophageal cancer.

## Results

### CD59 expression level was highly associated with radioresistance in the ESCC clinical specimens and cell lines

**Fig. 1:**
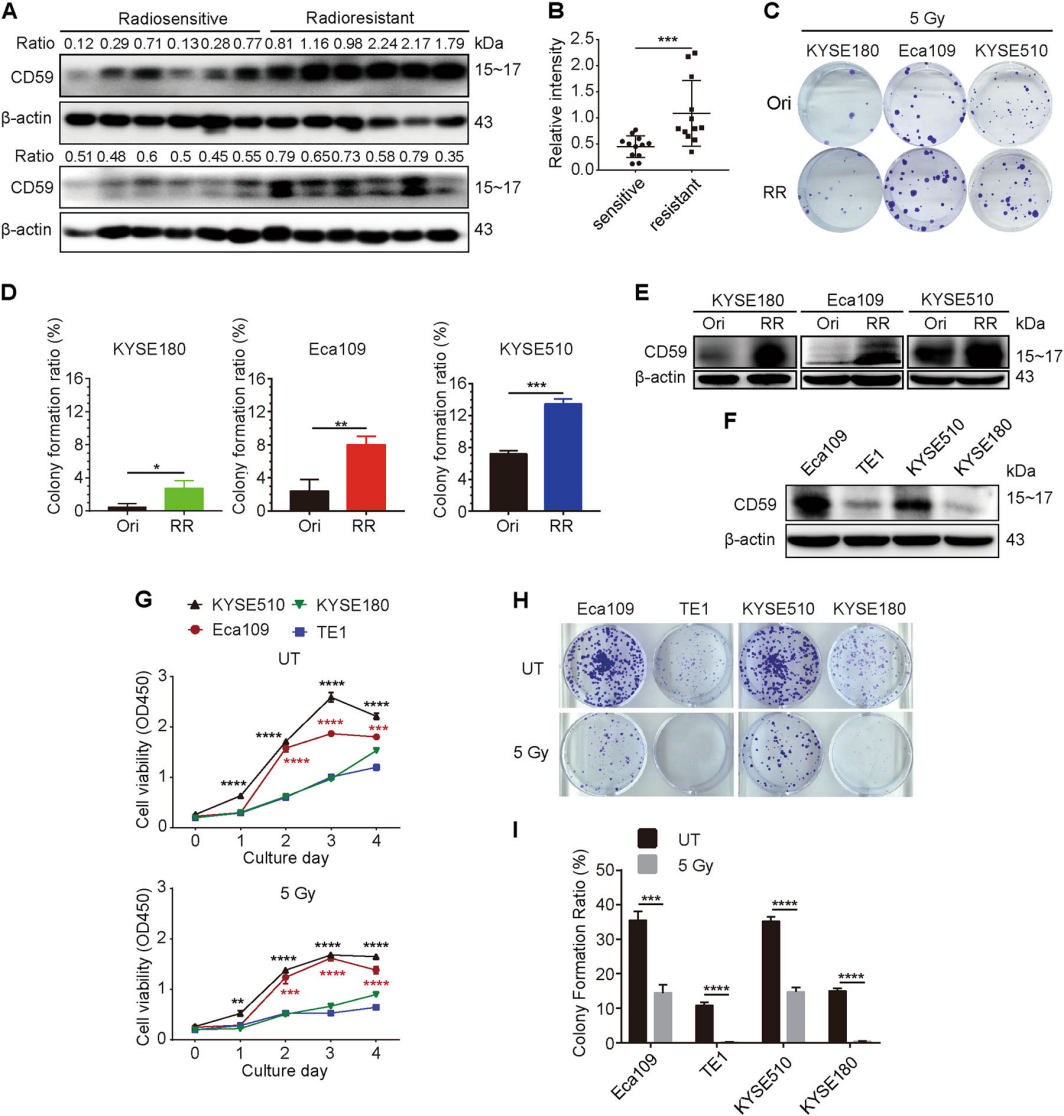
CD59 expression level is positively correlated with radioresistance in ESCC. **a** CD59 levels in radiosensitive specimens (left, *n* = 12) were significantly downregulated compared with those in radioresistant specimens (right, *n* = 12). All specimens were collected before radiotherapy from ESCC patients who received radiotherapy afterward. **b** Quantitative results from (**a**). **c**, **d** The colony formation ability of the radioresistant cells was significantly greater than that of the original cells after irradiation. The representative images in (**c**) and the quantitative results in (**d**). **e** CD59 was significantly upregulated in the three radioresistant cells compared with the expression in the corresponding original cells. **f** The comparison of CD59 expression levels in four original ESCC cell lines. **g** The dynamic growth of four ESCC cell lines with 5 Gy or without irradiation treatment (UT). KYSE510, KYSE180, or Eca109 vs TE1 cells. **h**, **i** The colony formation ability of four ESCC cell lines with or without irradiation. The representative images in h and the quantitative results in (**i**). RR: radioresistant cells to a total of 25 Gy (5 Gy 5 times). Data represent the mean ± SD, *n* = 3 unless otherwise indicated, *P < 0.05, **P < 0.01, ***P < 0.001 and ****P < 0.0001

To identify the relationship between CD59 expression and radiosensitivity in ESCC patients, we collected 24 ESCC clinical specimens before radiotherapy, of which half were classified as radiosensitive and half as radioresistant based on their recurrence after radiotherapy. The results showed that CD59 expression levels were significantly upregulated in the radioresistant ESCC tissues compared with the expression in the radiosensitive tissues (Figs. 1a, b). We further determined the correlation between CD59 expression and radioresistance in ECSS cell lines. The radioresistant ESCC cell lines were prepared by irradiation with 5 Gy γ-ray with 2-week intervals a total of five times. We observed that three resistant ESCC cell lines (KYSE180, Eca109, and KYSE510) were more resistant to 5 Gy γ-ray irradiation than their original cell lines, which was determined using the colony formation assay (Figs. 1c, d). Interestingly, CD59 expression was dramatically upregulated in these radioresistant ESCC cell lines (Fig. 1e). Furthermore, we compared the relationship between CD59 expression and radioresistance in four original ESCC cell lines. Eca109 cells expressed the much highest CD59, followed by KYSE510 cells, whereas TE1 and KYSE180 displayed weaker CD59 expression (Fig. 1f). The cell proliferation capability (Fig. 1g) and colony formation ability of the cell lines (Figs. 1h, i) before and after irradiation were ranked in the same order as the expression level of CD59. Together, these results suggested that the CD59 expression level was highly associated with radioresistance.

### Genetic alteration of CD59 expression affected the radioresistance of ESCC cells

**Fig. 2:**
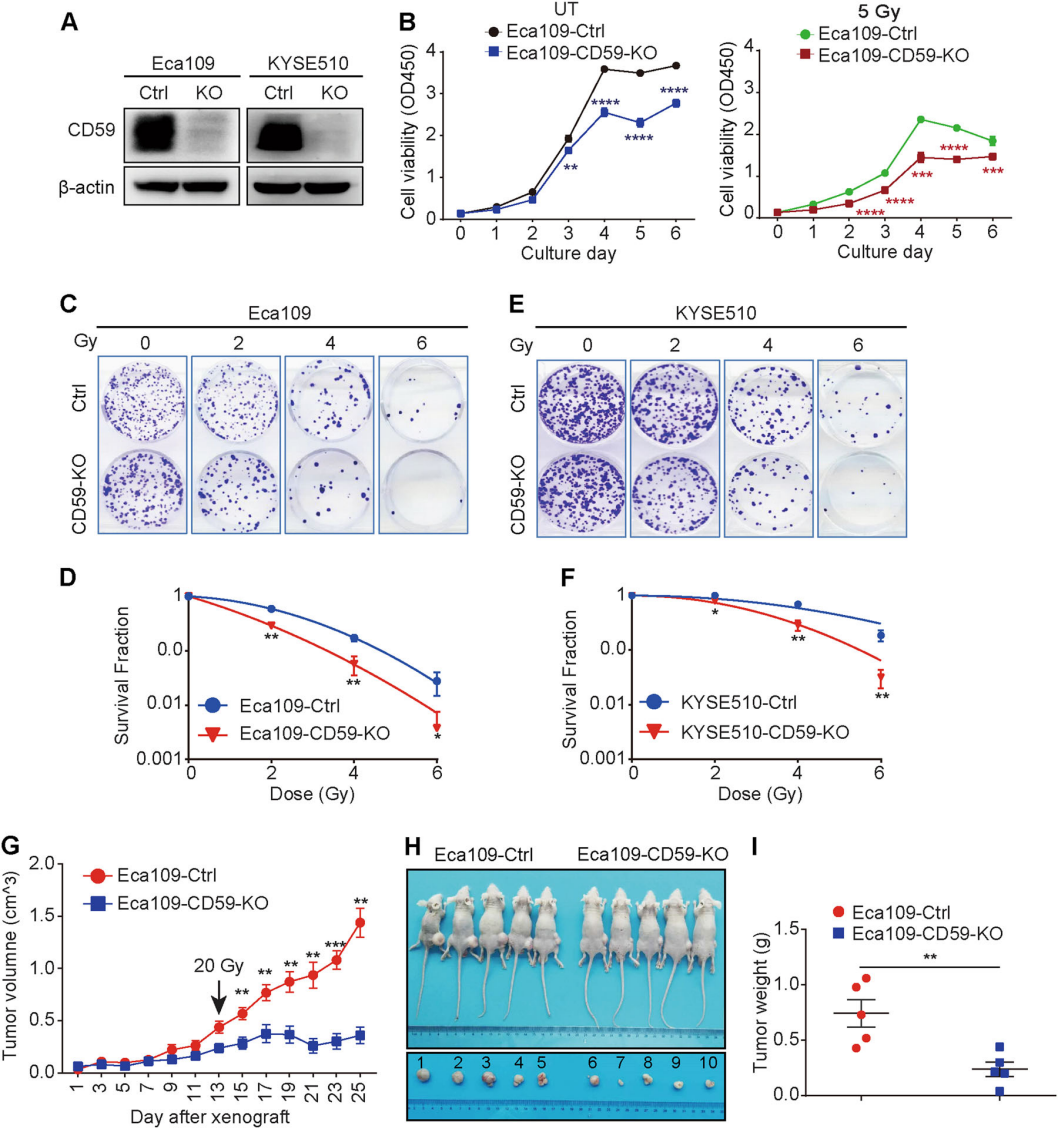
CD59 deficiency increases the radiosensitivity of ESCC. **a** The confirmation of CD59 deficiency. **b** CD59 deficiency suppressed the cell proliferation of Eca109 cells either with or without ionizing radiation. UT untreated. **c**–**f** CD59 deficiency impaired the colony formation ability of Eca109 (**c**, **d**) and KYSE510 (**e**, **f**) cells after ionizing radiation. The survival fraction was analyzed by a linear quadratic (LQ) model in (**d**) and (**f**). **g** CD59 deficiency significantly suppressed tumor growth compared with CD59 sufficiency in xenograft mice bearing Eca109 cells after irradiation treatment (*n* = 5). **h**, **i** The images of mice and corresponding tumors (**h**) and tumor weights (**i**) at the endpoint of the experiment (*n* = 5). Data represent the mean ± SD, *n* = 3 unless otherwise indicated, *P < 0.05, **P < 0.01, ***P < 0.001 and ****P < 0.0001

To further evaluate the impact of CD59 on radioresistance in ESCC, we first generated CD59 knockout cell lines, Eca109-CD59-KO and KYSE510-CD59-KO, using a CRISPR-Cas9 strategy, the efficacy of which was verified by western blot (Fig. 2a). CD59 deficiency significantly decreased cell proliferation in Eca109 cells either with (Fig. 2b, right panel) or without (Fig. 2b, left panel) irradiation. Furthermore, CD59 deficiency significantly increased the susceptibility of Eca109 cells to irradiation, in which the surviving clones were detected by crystal violet staining (Fig. 2c) followed by analysis of the survival fractions by a linear quadratic (LQ) model based on the colony formation results^46^ (Fig. 2d). Similar results were observed in KYSE510 cells (Figs. 2e, f). As expected, CD59 insufficiency in the radioresistant Eca109 cells induced by the specific short hairpin RNA (Fig. S1a) also significantly reduced radioresistance, which was determined by crystal violet staining and LQ model analysis for the surviving clones (Fig. S1b, c). By contrast, ectopic CD59 expression (Fig. S1d) significantly promoted cell proliferation either with (Fig. S1e, right panel) or without (Fig. S1e, left panel) irradiation in Eca109 cells. Moreover, ectopic CD59 expression also reduced the susceptibility of Eca109 cells to irradiation (Fig. S1f, g).

We next confirmed the function of CD59 on increasing radiosensitivity in vivo. The CD59-sufficient or -deficient Eca109 cells were implanted into Balb/c nude mice, which were treated with a single dose of 20 Gy γ-rays on day 13 when the tumor volume reached 0.3 – 0.5 cm^3^. We observed that the CD59-deficient tumors were significantly more sensitive to irradiation than the CD59-sufficient tumors, demonstrated by tumor growth (Fig. 2g), tumor images (Fig. 2h), and tumor weight (Fig. 2i) at the endpoint of the experiment. Irradiation treatment could trigger the strong complement activation in the tumor-bearing mice, probably resulting from the irradiation-induced cell death and the increased C3 expression^27^. Considering the important role of CD59 in restricting MAC formation during complement activation, we collected the above tumor tissues and detected the MAC deposition by immunohistochemistry (IHC). The results showed that MAC was extensively stained in a comparable level between control and CD59-KO Eca109 cells (Fig. S2). Therefore, these results suggest that genetic alteration of CD59 expression significantly affected the radioresistance of ESCC cells most likely in a complement-independent manner.

### CD59 deficiency impaired DNA damage repair of ESCC cells after irradiation

**Fig. 3:**
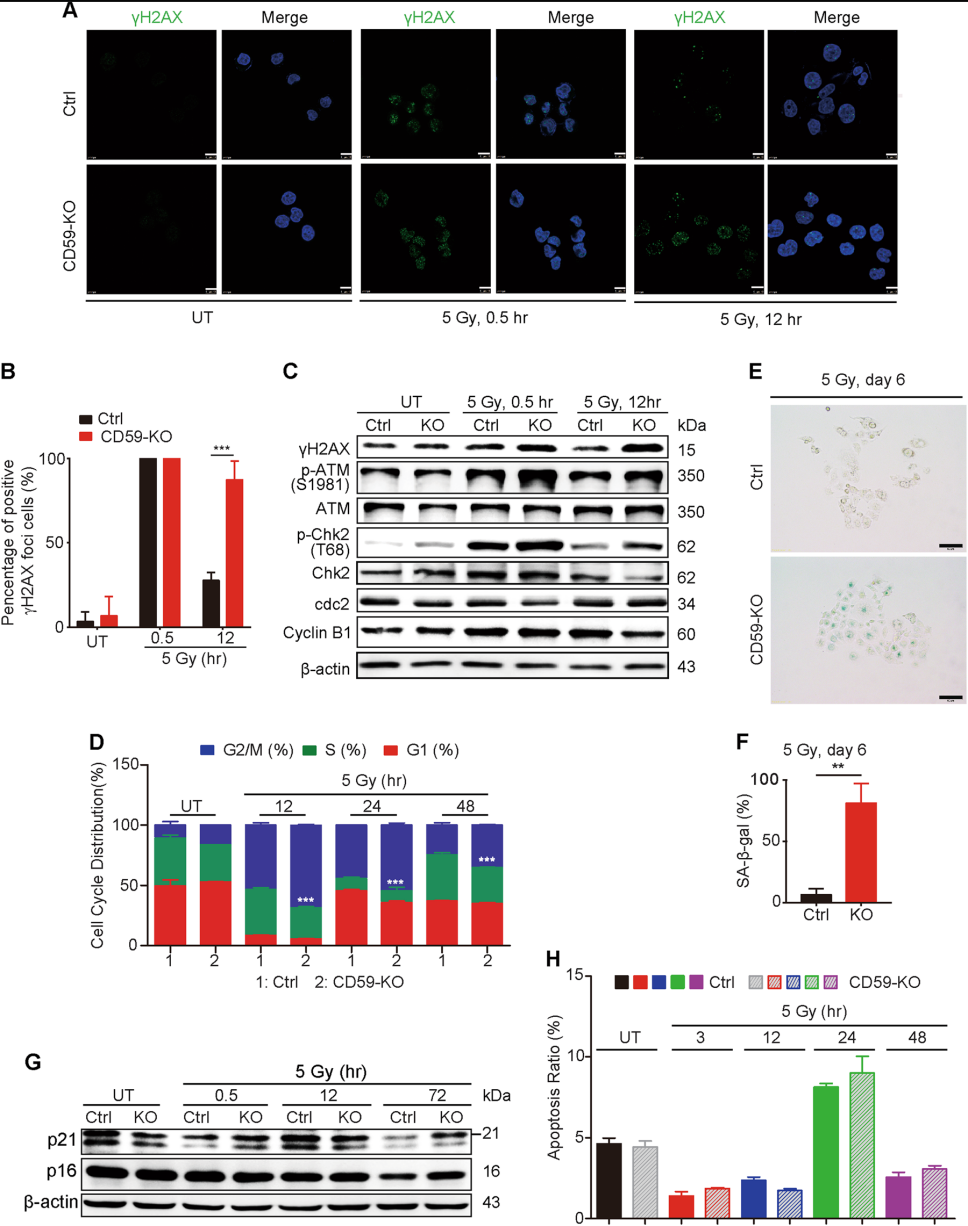
CD59 deficiency impairs DNA damage repair after irradiation. **a**, **b** The formation of γH2AX foci in the CD59-deficient Eca109 cells significantly increased compared with that in CD59-sufficient cells at 12 h after ionizing radiation. The representative images for γH2AX foci in (**a**) and the quantitative results in (**b**) for measuring the percentage of γH2AX-positive ( > 10 foci/cell) cells. Scale bar, 10 μm. **c** The expression or phosphorylation levels of the DDR-related signaling molecules were significantly changed upon irradiation in Eca109 cells, which was further enhanced by CD59 deficiency. **d** CD59 deficiency exacerbated G2/M phase arrest compared with CD59 sufficiency in Eca109 cells upon irradiation treatment. **e**, **f** CD59 deficiency induced greater cellular senescence in Eca109 cells after irradiation than CD59 sufficiency as accessed by SA-β-gal (Senescence-associated beta-galactosidase) staining. The representative images in (**e**) and the quantitative results in (**f**). **g** CD59 deficiency induced a smaller decrease in the levels of p21 and p16 than CD59 sufficiency in Eca109 cells after irradiation. **h** Irradiation-induced apoptosis of Eca109 cells at 24 h; however, there was no significant difference between CD59-sufficient and -deficient cells. Data represent the mean ± SD, *n* = 3, **P < 0.01 and ***P < 0.001

The development of γH2AX foci is a sensitive marker for DSBs^7,47^. Thus, we detected γH2AX levels to evaluate the DNA damage/repair response to CD59 deficiency after irradiating Eca109 cells. Using IHC staining, we failed to detect γH2AX foci before irradiation in both CD59-sufficient and -deficient cells (Fig. 3a, left panel). The γH2AX foci-positive cells ( > 10 foci/cell) reached a peak at 0.5 h after irradiation; however, there were no significant differences between the CD59-sufficient and -deficient cells (Fig. 3a, middle panel). At 12 h after irradiation, the number of γH2AX foci-positive cells significantly decreased in CD59-sufficient cells compared with that in CD59-deficient cells (Fig. 3a, right panel). The related quantitative results are shown in Fig. 3b. The detection of γH2AX and levels of its upstream molecule phospho-ATM by western blot also supported the above results (Fig. 3c), indicating that CD59 deficiency impaired the DNA repair process because γH2AX foci disappear after DSBs are repaired^47^.

ATM is activated in response to DSBs induced by ionizing radiation, leading to checkpoint arrest and the accumulation of repair proteins at DSB sites via downstream pathways after γH2AX foci formation^47^. ATM phosphorylates Chk2 and rapidly inactivates the cyclin B-cdc2 complex, preventing cells with genomic DNA damage from entering mitosis (M phase) via G2/M checkpoint arrest, thus allowing time for DNA damage repair. Upon ionizing radiation, the Chk2 phosphorylation level was rapidly and significantly increased at 0.5 h and decreased to baseline at 12 h in both CD59-sufficient and -deficient Eca109 cells (Fig. 3c). Furthermore, CD59-deficient cells exhibited higher Chk2 phosphorylation levels at 0.5 and 12 h than CD59-sufficient cells, resulting in downregulated levels of cdc2 at 0.5 h and cyclin B1 at 12 h (Fig. 3c). Next, we functionally detected the effect of CD59 deficiency on cell cycle arrest after irradiation. The results showed that significant cell cycle arrest at the G2/M phase occurred at 12, 24, and 48 h post-irradiation in CD59-deficient cells compared with that in CD59-sufficient cells (Fig. 3d).

If cell cycle arrest continues and DNA damage becomes irreparable, cellular senescence may subsequently be induced^13^. Therefore, we next detected the effect of CD59 expression on the occurrence of senescence at day 6 after ionizing radiation by SA-β-gal staining. The results revealed that CD59 deficiency significantly increased the percentage of senescent Eca109 cells upon irradiation compared with that observed in CD59-sufficient Eca109 cells (Figs. 3e, f). The expression levels of the associated signaling molecules p21 and p16 significantly decreased at 72 h after irradiation in both CD59-sufficient and -deficient cells compared with the levels in the corresponding untreated cells, in which CD59-sufficient cells showed a greater decrease in p21 and p16 expression than CD59-deficient cells (Fig. 3g). Apoptosis also involves an antitumor effect induced by irradiation;^13^ therefore, we detected the apoptotic effect in CD59-sufficient and deficient cells after ionizing radiation. The results showed that irradiation slightly enhanced apoptosis at 24 h after treatment in both CD59-sufficient and -deficient cells; whereas CD59 deficiency failed to potentiate apoptosis (Fig. 3h), indicating different mechanisms between cell cycle arrest and apoptosis induced by ionizing radiation in ESCC cells. Therefore, these findings suggested that CD59 deficiency may suppress cell proliferation via G2/M phase checkpoint arrest and further cellular senescence, thus enhancing radiosensitivity.

### CD59 deficiency reduced the phosphorylation of p-Src (Y416), and a Src inhibitor sensitized ESCC cells to irradiation

**Fig. 4:**
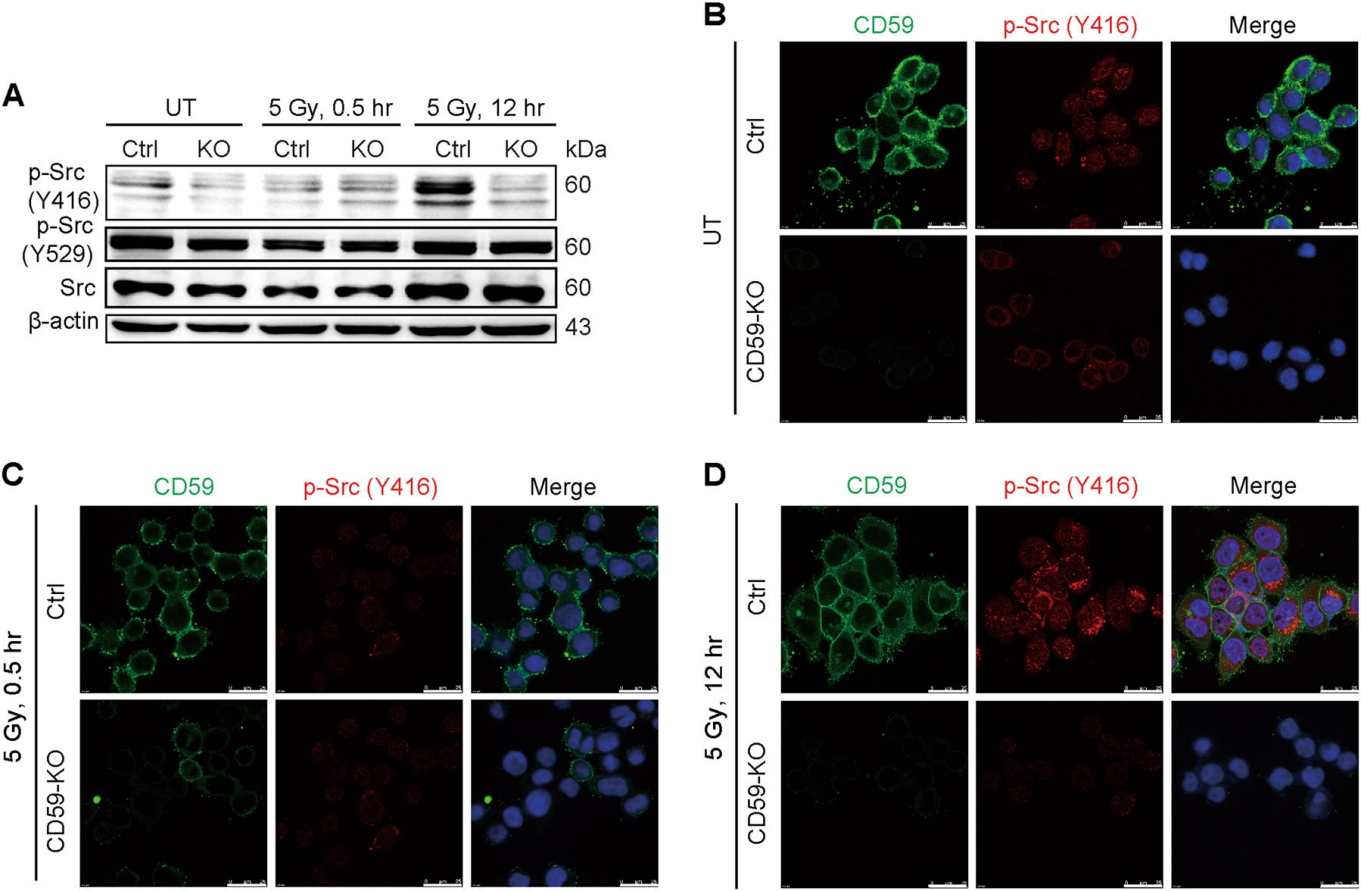
CD59 deficiency reduces the phosphorylation of Src (Y416). **a**-**d** CD59 deficiency resulted in the failure of Src activation after irradiation. Irradiation induced the activation of Src at 12 h in CD59-sufficient Eca109 cells, which was determined by the increased phosphorylation of the active site Y416 (**a**-**d**) but not that of inhibitory site Y529 (**a**). However, Src remained inactive in CD59-deficient cells either with or without irradiation (**a**-**d**). The western blot assay in (**a**) and the immunofluorescence assay in (**b**-**d**)

The above in vitro results that CD59 expression highly correlated with radioresistance suggested that CD59 played a complement-independent role in DDR. Considering the involvement of CD59 in the formation of lipid rafts enriched with Src kinase family members^39–43^, we next detected the activation of Src in CD59-sufficient and -deficient Eca109 cells. Either with or without irradiation, the total Src expression level and the inhibitory phosphorylation of Src (Y529) were not affected by CD59 deficiency compared with CD59 sufficiency; however, the active phosphorylation of Src (Y416) was significantly increased only in CD59-sufficient cells at 12 h after irradiation compared with that at 0.5 h after irradiation and without irradiation (Fig. 4a). Notably, Src phosphorylation (Y416) in CD59-deficient cells was unresponsive to irradiation, with Src remaining inactivated both without irradiation and with irradiation after 0.5 and 12 h (Fig. 4a). Moreover, the immunofluorescence assay showed similar results for the phosphorylation of Src (Y416) (Figs. 4b-d). These results showed that CD59 deficiency significantly affected the activation of Src at Y416 after irradiation.

**Fig. 5:**
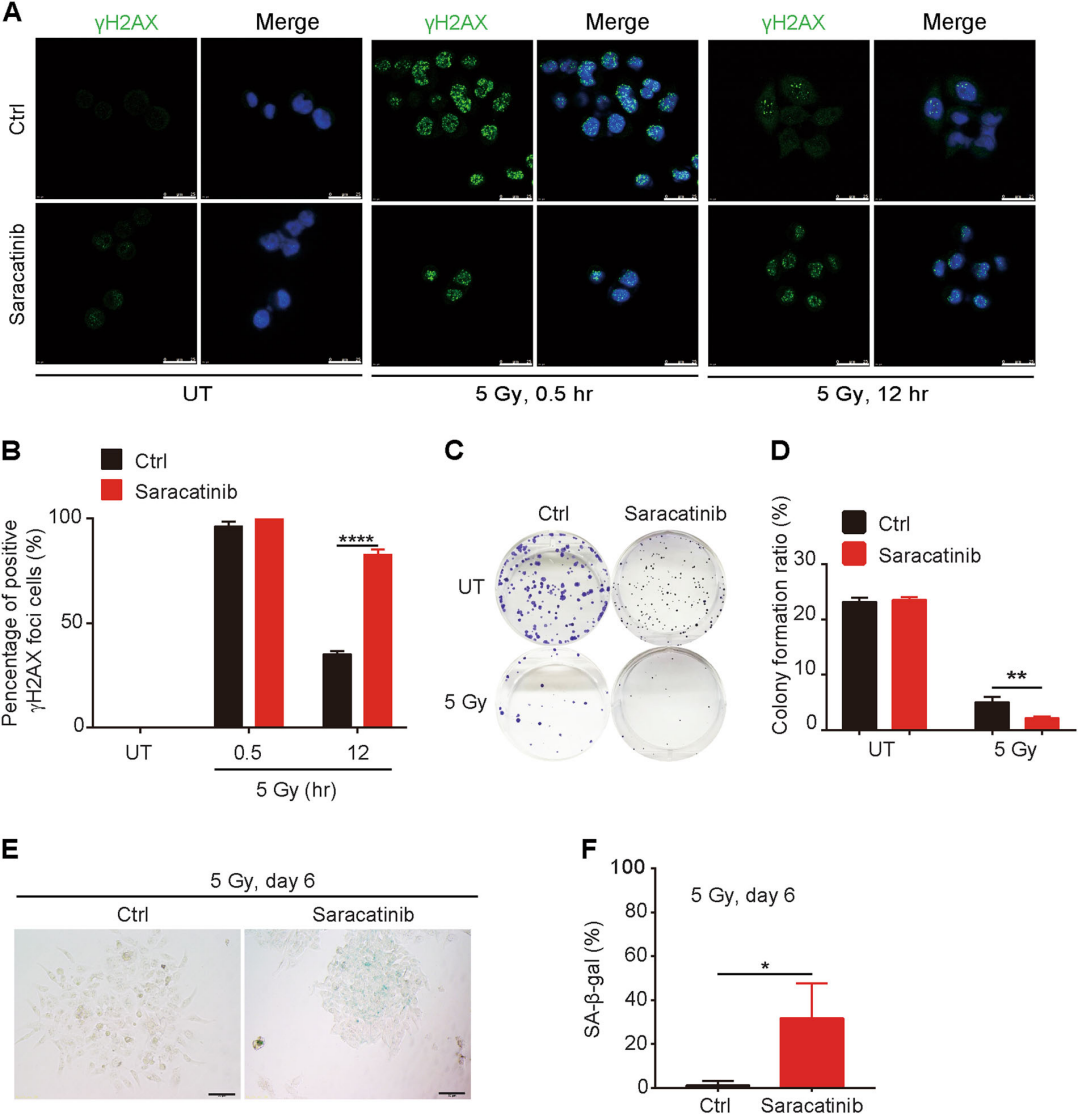
Src inhibition potentiates the susceptibility of ESCC to irradiation. **a**, **b** Saracatinib significantly increased the formation of γH2AX foci in Eca109 cells at 12 h after ionizing radiation. The representative images for γH2AX foci in (**a**) and the quantitative results in (**b**) for measuring the percentage of γH2AX-positive ( > 10 foci/cell) cells. Scale bar, 25 μm. **c**, **d** Saracatinib impaired the colony formation ability of Eca109 cells after irradiation. The representative images in (**c**) and the quantitative results in (**d**). **e**, **f** Saracatinib enhanced the cellular senescence induced by irradiation. The representative images in (**e**) and the quantitative results in (**f**). Cellular senescence was detected by SA-β-gal staining. Data represent the mean ± SD, *n* = 3, *P < 0.05, **P < 0.01 and ****P < 0.0001

We further detected whether chemical inhibition of Src may enhance the susceptibility of ESCC cells to irradiation. Similar to CD59 deficiency, saracatinib, a Src inhibitor, significantly increased DNA damage upon irradiation, which was demonstrated by significantly increased accumulation of γH2AX foci compared with that observed after control treatment, particularly at 12 h after irradiation (Figs. 5a, b). Saracatinib also reduced the colony formation ability of Eca109 cells after irradiation (Figs. 5c, d). Notably, although saracatinib significantly suppressed the growth of colonies represented by their smaller size compared with the control treatment, it failed to inhibit the colony formation ability of Eca109 cells without irradiation (Fig. 5c). Moreover, saracatinib significantly induced Eca109 cell into senescence. The percentage of senescent cells was significantly increased by saracatinib treatment (30.4%) compared with that in control treatment (3.1%) at 6 days after irradiation (Figs. 5e, f). Together, these results indicated that CD59 deficiency reduced the phosphorylation of p-Src (Y416), subsequently potentiating the susceptibility of ESCC cells to ionizing radiation.

### CD59 expression levels were closely associated with the prognosis of ESCC patients

**Fig. 6:**
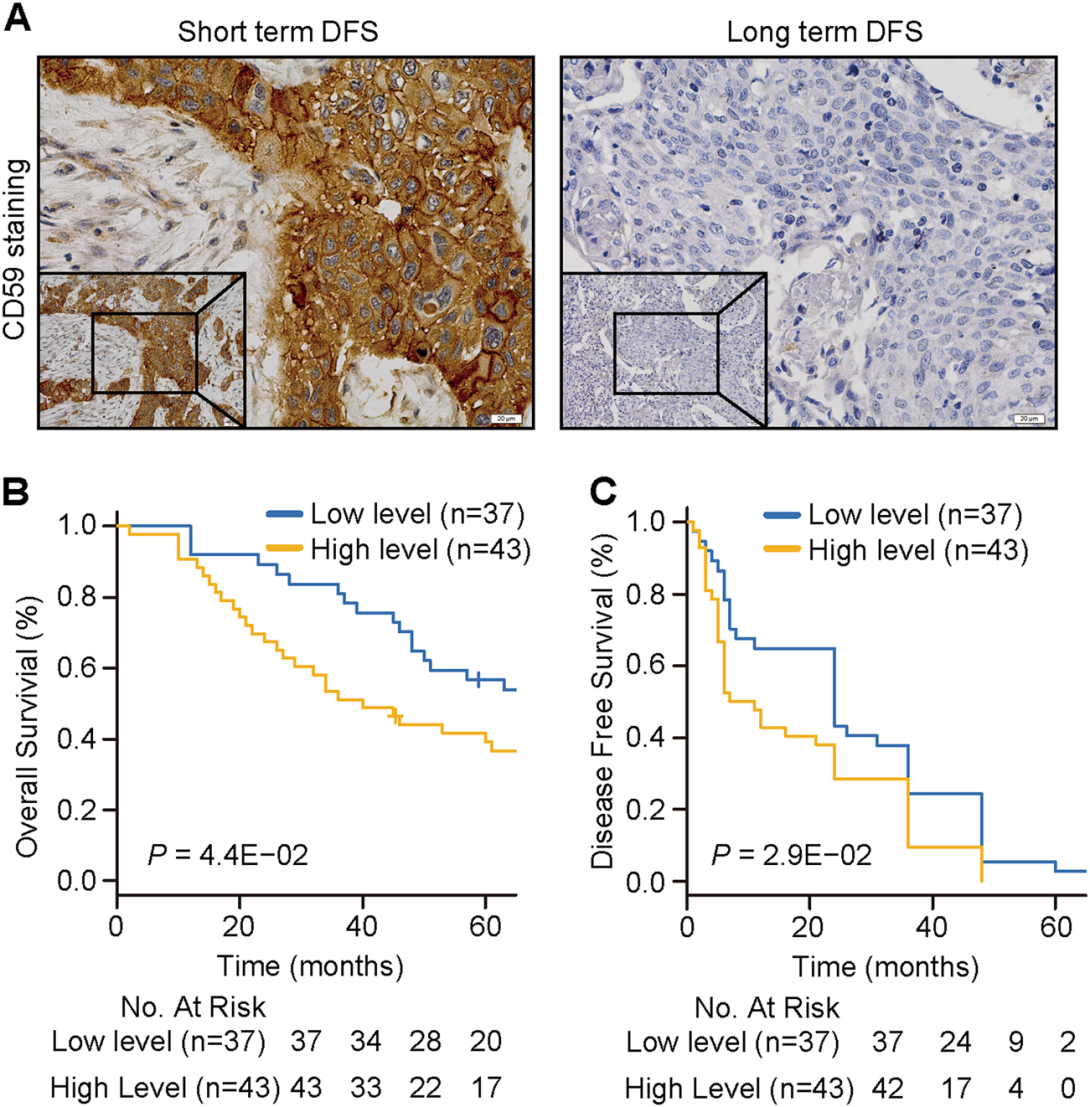
The CD59 level is highly correlated with the prognosis of ESCC patients receiving radiotherapy. **a** The representative images of CD59-positive (left) and -negative (right) staining in clinical specimens before radiotherapy from ESCC patients receiving radiotherapy afterward. Short-term DFS associated with positive CD59 staining and long-term DFS associated with negative CD59 staining. Scale bar, 20 μm. **b**, **c** Kaplan-Meier survival curves of OS (**b**) and DFS (**c**) based on CD59 expression levels in 80 cancer tissues. OS overall survival, DFS disease-free survival

To investigate the relationship between CD59 expression levels and the prognosis of ESCC patients, we collected 80 specimens before radiotherapy from different ESCC patients who received radiotherapy afterward. The related clinicopathological features are shown in Table 1. The expression levels of CD59 were detected by IHC with specific antibodies, and representative images with positive or negative CD59 staining are shown in Fig. 6a. There was no statistical correlation between CD59 expression levels and age, gender, or tumor stage of the patients (Table 1). However, patients with a high CD59 level displayed shorter OS and disease-free survival (DFS) after radiotherapy than those with a low CD59 level (Figs. 6b, c). These results indicated that CD59 has a great potential as a prognostic indicator and is possibly a radiotherapy target for ESCC.

**Table 1:** The association of the expression of CD59 with the clinicopathological features from ESCC patients (*n* = 80)

	CD59		*P*
	Low level	High level	
Age			0.303
<60	24	23	
≥60	13	20	
Gender			0.363
Male	30	38	
Female	7	5	
Stage			0.713
I–II	17	18	
III–IV	20	25

## Discussion

Radiation is an important modality for esophageal cancer therapy. Acquisition of radioresistance ultimately results in relapse and failure of therapy. In this study, we revealed that the expression levels of CD59 in ESCC cell lines and clinical specimens was positively correlated with the radioresistance of ESCC. High CD59 levels predicted a poor prognosis in ESCC patients. CD59 deficiency increased the radiosensitivity of ESCC, whereas ectopic CD59 expression exerted the opposite effect. Furthermore, the effect of CD59 on modulating radiosensitivity was probably mediated via Src molecules in lipid rafts. These results suggest that CD59 potentially provides a biomarker for predicting the radiosensitivity and prognosis in ESCC patients.

Induction of DNA damage is the hallmark of the radioresponse^5,6^, which further induces the DDR. The DDR pathway consists of sensors, transducers and effectors of DNA damage^48,49^. The MRE11-RAD50-NBS1 (MRN) complex detects DSBs and recruits ATM to promote ATM-mediated γH2AX, which surrounds the DNA lesions and forms visualized foci^7,50^. ATM can also induce cell cycle arrest, mainly via Chk2, allowing time for the repair of DSBs^51^. Subsequently, a number of signaling and repair proteins accumulate at DNA lesions and form discrete foci^52,53^. The removal of γH2AX was shown to be an indicator of the completion of DNA repair^47,54^. We demonstrated that CD59 deficiency in ESCC cells hindered the removal of γH2AX foci until 12-h post-irradiation. Consistent with this, the phosphorylation levels of ATM (S1981) and Chk2 (T68) were upregulated, thus leading to reduced levels of cdc2 and cyclin B1 in CD59-deficient ESCC cells after ionizing radiation. All these factors further induced significant G2/M arrest. Therefore, CD59 deficiency resulted in defective DDR, which resulted in excessive unrepaired DNA damage and prolonged G2/M arrest.

The irreversible state of cell cycle arrest may further result in cellular senescence^55^. Premature senescence can act as a tumor-suppressor mechanism that removes cells harboring DNA damage from the proliferative state^56^, whereas stress-induced senescence results from exposure to endogenous or exogenous signals, such as radiation or chemotherapy^57^. We revealed that CD59 deficiency significantly induced cellular senescence in ESCC cells after ionizing radiation, most likely due to increased levels of p21 and p16^58,59^. In addition, unrepaired DNA damage may induce cell death via both senescence and apoptosis^60^. Although we observed approximately 10% apoptotic cells at 24 h after ionizing radiation in both CD59-sufficient and -deficient ESCC cell, there was no significant difference between the two subpopulations. This result suggests that apoptosis may not be the main mechanism for radiosensitivity in CD59-deficient ESCC cells.

As an important complement regulator protein, CD59 mainly restricts the assembly of MAC in the complement-targeted cell membrane. We found that the colony formation ability of CD59-deficient cells upon irradiation was significantly reduced in complement-inactivated serum, indicating a complement-independent role of CD59 in modulating the response of ESCC cells to ionizing radiation. In addition, the MAC displayed an extensive and similar staining in tumor tissues derived from control and CD59-KO esophageal cancer cells, which further supports the complement-independent role of CD59 in modulating radiosensitivity. CD59 couples with Src-family kinases to mediate signaling transduction^39,41–43,61^. Here, we observed that phospho-Src (Y416) was highly activated in CD59-sufficient Eca109 cells at 12-h post-irradiation, whereas CD59 deficiency significantly reduced Src activation despite irradiation. Src kinase activity was reported to be associated with the efficacy of chemotherapy and radiotherapy. Src expression strongly inhibits adriamycin-induced senescence and G2 checkpoint arrest by blocking the induction of p21, thus leading to the chemo-resistance of fibrosarcoma cells^62^. Src activity was increased in lapatinib-resistant breast cancer cells, and the Src inhibitor saracatinib in combination with lapatinib restored the sensitivity of cells resistant to lapatinib^63^. Furthermore, a Src inhibitor has also been shown to enhance radiosensitivity of malignant glioma cells^64^ and lung cancer cells^65^. We found that saracatinib increased the susceptibility of ESCC cells to ionizing radiation by elevating DNA damage, reducing colony formation, and inducing cellular senescence. Therefore, CD59 deficiency-induced radiosensitivity likely results from weakened Src signaling.

## Materials and methods

### ESCC tissue samples

Eighty paraffin-embedded ESCC tissue samples from radiotherapy patients were collected for IHC staining. Twenty-four fresh ESCC tissue samples were collected during surgery before radiotherapy and frozen at −80 °C for western blotting analysis. Among the 24 patients providing fresh samples, 12 radiosensitive patients were classified by smaller tumor size and without local recurrence beyond 1 year after radiotherapy, and the other 12 radioresistant patients were classified by unchanged or larger tumor size and with local recurrence within half a year after radiotherapy. The Ethics Committee at Fudan University Shanghai Cancer Center approved the utilization of samples, and all patients signed the informed consent form.

### ESCC cell lines and treatment

Four human ESCC cell lines, KYSE510, KYSE180, Eca109, and TE1, were cultured in RPMI-1640 medium (HyClone, South Logan, UT) supplemented with 10% fetal bovine serum (Gibco, Thermo Fisher Scientific, Waltham, MA) and 5% penicillin–streptomycin antibiotics (Gibco, Thermo Fisher Scientific, Waltham, MA). The ^137^Cs gamma-ray irradiation apparatus Gamma-cell ®40 Exactor (Nurdion, Canada) was used to generate γ-rays for in vitro and in vivo experiments. The Src inhibitor saracatinib (AZD0530) was purchased from Selleck Chemicals (Houston, TX, USA).

### Xenograft mouse model

The study was approved by the Animal Ethics Committee at Shanghai Medical School, Fudan University. The 6-week-old Balb/c nude mice (*n* = 5) were subcutaneously injected with Eca109-Ctrl and Eca109-CD59-KO cells. The tumor site received a single dose of 20 Gy γ-rays when the tumor volume reached approximately 0.3 – 0.5 cm^3^. The tumor volumes were measured every 2 days and calculated as L × W^2^ × 0.5 (cm^3^), in which L and W represent the longest and shortest diameter, respectively. Fifteen days post-irradiation, the mice were anesthetized and the tumors were harvested and weighed.

### Construction of CD59 knockout and overexpression stable cell lines

The CRISPR/Cas9 technique was utilized to construct CD59 knockout ESCC cell lines. The pLenti-Cas9-blast and pgRNA-puromycin vectors were kind gifts from Dr. Shenglin Huang (Fudan University Shanghai Cancer Center). The coding sequence (CDS) of CD59 was amplified by PCR, inserted into the lentivirus vector pCDH-puro and packaged into lentivirus. The ESCC cell lines stably expressing CD59 were obtained by lentivirus infection and screening with puromycin at a concentration of 10 μg/mL.

### IHC assay

MAC deposition in tumor tissues of the implanted mice was detected by IHC using anti-C5b-9n antibody (Abcam, Cambridge, MA, USA). CD59 expression in the ESCC clinical samples was detected by IHC staining using an anti-CD59 antibody (Abcam, Cambridge, MA, USA). Briefly, slices were deparaffinized in xylol. Antigen retrieval was performed using 10 mmol/L sodium citrate (pH 6.0), endogenous peroxidase activity was inhibited with 3% hydrogen peroxide, and the slices were blocked with 1% bovine serum albumin/phosphate-buffered saline. Slices were placed in a humidified chamber and incubated with anti-CD59 antibody at 4 °C overnight. Reactions were developed using GTvision TM III (Gene Technology, Shanghai, China) and counterstained with 10% hematoxylin. Finally, slices were dehydrated and mounted with resinene. The staining index (0–12) for CD59 was obtained as the staining intensity (negative (0); weak (1); moderate (2); and strong (3)) multiplied by the proportion of positive staining ((0–25% (1); 25–50% (2); 50–75% (3); and 75–100% (4))^66,67^. Two experienced pathologists blinded to the clinical data scored the staining results.

### Western blot analysis

We performed western blotting according to a standard protocol. The antibodies against the indicated proteins were purchased from Abcam (Cambridge, MA, USA), Cell Signaling Technology (Danvers, MA, USA), and Santa Cruz Biotechnology (Santa Cruz, CA, USA). The catalogs were listed as follows: CD59 (ab9183, Abcam), Src (ab109381, Abcam), p-Src (Y416) (#6943, Cell Signaling Technology), p-Src (Y529) (ab32078, Abcam), ATM (#2873, Cell Signaling Technology), p-ATM (Ser1981) (#5883, Cell Signaling Technology), p-CHK2 (Thr68) (#2197T, Cell Signaling Technology), cyclin B1 (sc-245, Santa Cruz), γH2AX (Ser139) (ab22551, Abcam), NBS1 (#3002, Cell Signaling Technology), p16 (ab81278, Abcam), p21 (ab109199, Abcam), and β-actin (sc-7963, Santa Cruz).

### Survival fraction

A LQ model was applied to analyze the survival fraction of esophageal cancer cells after ionizing radiation^68^.

### Cell proliferation and colony formation assays

For the cell proliferation assay, cells were seeded into 96-well plates at a density of 1500 cells/well and treated with or without irradiation. The cell proliferation curves were constructed according to the OD450 value tested by the Cell Counting Kit-8 (CCK8, Dojindo, Kumamoto, Japan). For the colony formation assay, cells were seeded into six-well plates at density of 500 or 1000 cells/well (Fig. 1h) and treated with and without irradiation the next day. Two weeks later, the medium was discarded, and the cells were fixed with 4% paraformaldehyde and stained with 0.5% crystal violet. Colonies were counted, and the colony formation ratio was calculated as follows: colony formation ratio = (colonies formed/cells seeded) × 100%.

### Statistical analysis

Statistical evaluation was conducted with SPSS 22.0 (SPSS Inc., Chicago, IL). The chi-square test was used to analyze the relationship between clinical pathological parameters and the expression level of CD59. The 5-year OS and DFS were calculated using the Kaplan–Meier method, and differences in variables were compared using log-rank tests. The significance of the in vitro and in vivo data was determined using Student’s *t*-test (two-tailed). All data were shown as the mean ± SD, and a *P*-value <0.05 was considered significant.

## Electronic supplementary material

Supplementary figure 1

Supplementary figure 2

Supplementary figure legends
