# Supplementary figures and images for: CD59 is a potential biomarker of esophageal squamous cell carcinoma radioresistance by affecting DNA repair
Source: Cell Death Dis. 2018 Aug 30;9(9):887. doi: 10.1038/s41419-018-0895-0 (PMC6117325; doi:10.1038/s41419-018-0895-0)

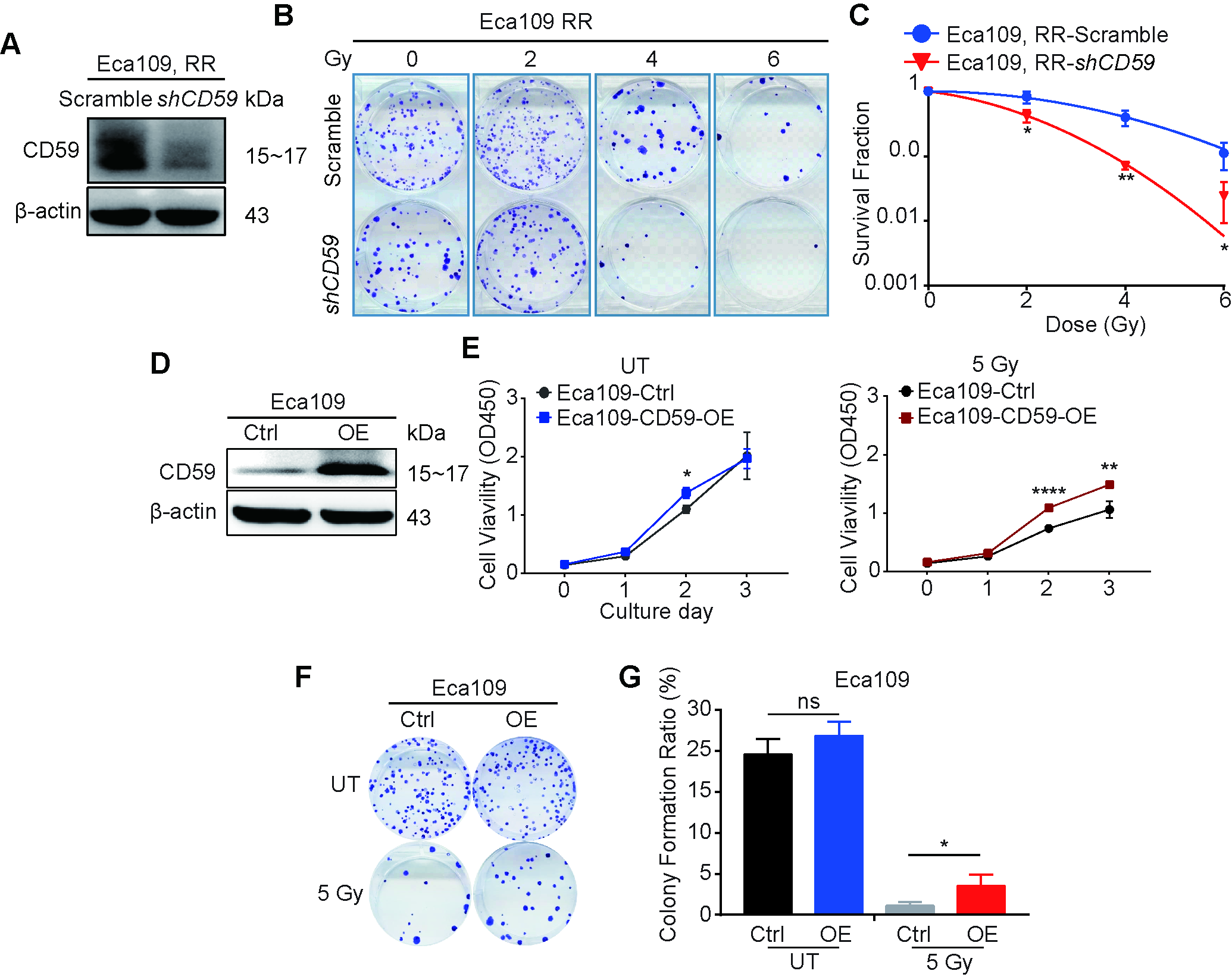

Supplement: Supplementary file 1 — Supplementary figure 1 [file 41419_2018_895_MOESM1_ESM.tif]

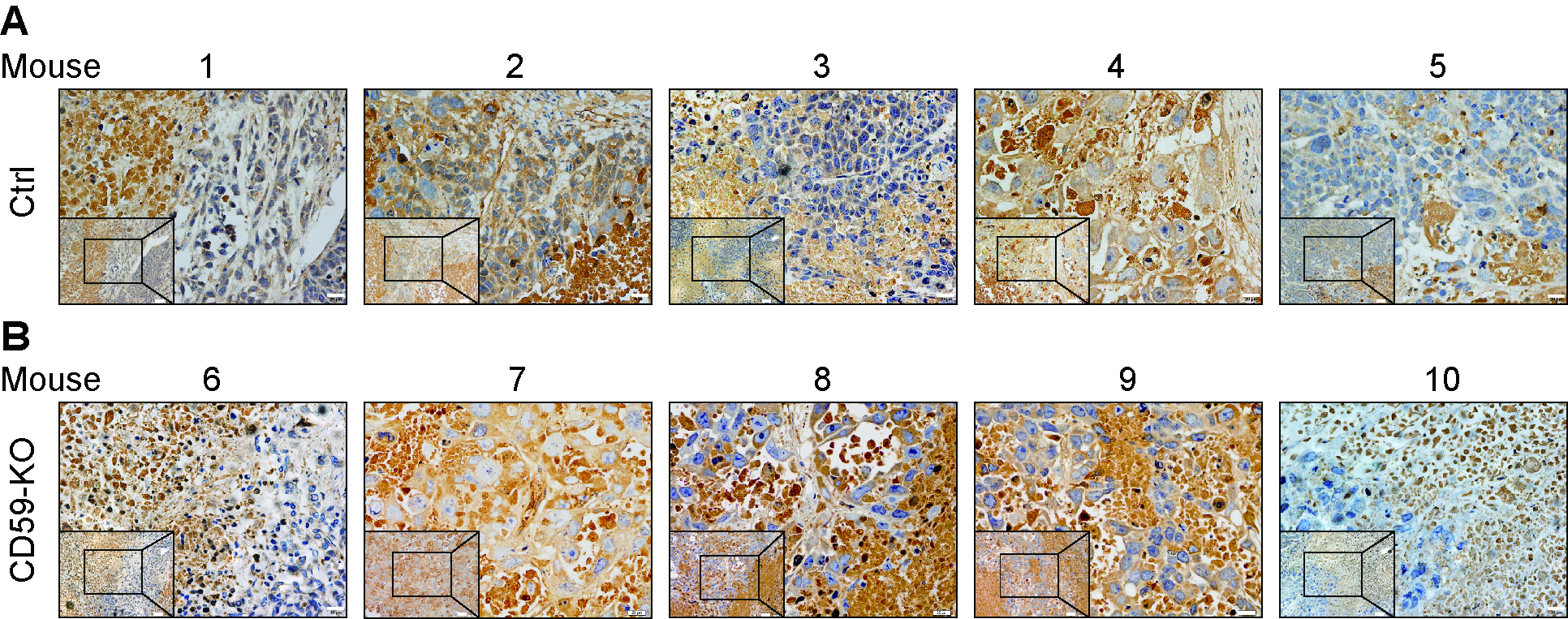

Supplement: Supplementary file 2 — Supplementary figure 2 [file 41419_2018_895_MOESM2_ESM.tif]
