# Supplementary material for: CD59 is a potential biomarker of esophageal squamous cell carcinoma radioresistance by affecting DNA repair
Source: Cell Death Dis. 2018 Aug 30;9(9):887. doi: 10.1038/s41419-018-0895-0 (PMC6117325; doi:10.1038/s41419-018-0895-0)
Supplement: Supplementary file 3 — Supplementary figure legends [file 41419_2018_895_MOESM3_ESM.docx]

**Supplementary Figure legend**

**Supplementary Fig. S1 Genetic alteration of CD59 expression affects the susceptibility of Eca109 cells to ionizing radiation.**

a The efficacy of CD59 knockdown by the specific shRNA in Eca109 cells. b and c CD59 insufficiency suppressed the colony formation ability of radioresistant Eca109 cells after irradiation. The representative images in (b) and the survival fraction analyzed by the LQ model in (c). d Confirmation of ectopic CD59 expression in Eca109 cells. e and f Ectopic CD59 expression promoted the cell proliferation of Eca109-CD59 cells with (F) but not without (e) ionizing radiation compared to normal CD59 expression. g and h Ectopic CD59 expression increased the colony formation ability of Eca109 cells with but not without ionizing radiation compared to normal CD59 expression. The representative images in (g) and the quantitative results in (h). UT, untreated. Data represent the mean ± SD, n=3, * P<0.05, ** P<0.01, and **** P<0.0001.

**Supplementary Fig. S2 Complement is extensive activated in a comparable level in both mouse models implanted with control (A) or CD59-KO (B) Eca-109 esophageal cells**. The complement activation was determined by MAC deposition, which was stained by anti-C5b-9n antibody. The mouse number is in line with that in Fig. 2h. Scale bar, outside box 20 μm and inside box 50 μm.
